# How mathematical modeling could contribute to the quantification of metastatic tumor burden under therapy: insights in immunotherapeutic treatment of non-small cell lung cancer

**DOI:** 10.1186/s12976-021-00142-1

**Published:** 2021-06-02

**Authors:** Pirmin Schlicke, Christina Kuttler, Christian Schumann

**Affiliations:** 1 Center of Mathematics, Technical University of Munich, Boltzmannstraße, Garching, Germany; 2 Clinic of Pneumology, Thoracic Oncology, Sleep and Respiratory Critical Care, Klinikverbund Allgäu, Robert-Weichsler-Straße, Kempten, Germany

**Keywords:** Metastatic tumors, Treatment, Chemotherapy, Immunotherapy

## Abstract

**Background:**

Cancer is one of the leading death causes globally with about 8.2 million deaths per year and an increase in numbers in recent years. About 90% of cancer deaths do not occur due to primary tumors but due to metastases, of which most are not clinically identifiable because of their relatively small size at primary diagnosis and limited technical possibilities. However, therapeutic decisions are formed depending on the existence of metastases and their properties. Therefore non-identified metastases might have huge influence in the treatment outcome. The quantification of clinically visible and invisible metastases is important for the choice of an optimal treatment of the individual patient as it could clarify the burden of non-identifiable tumors as well as the future behavior of the cancerous disease.

**Results:**

The mathematical model presented in this study gives insights in how this could be achieved, taking into account different treatment possibilities and therefore being able to compare therapy schedules for individual patients with different clinical parameters. The framework was tested on three patients with non-small cell lung cancer, one of the deadliest types of cancer worldwide, and clinical history including platinum-based chemotherapy and PD-L1-targeted immunotherapy. Results yield promising insights into the framework to establish methods to quantify effects of different therapy methods and prognostic features for individual patients already at stage of primary diagnosis.

## Background

The vast majority of primary malignant lung tumors are carcinomas, which contain two major subgroups: non-small cell lung cancer (NSCLC) and small cell lung cancer (SCLC). The former group accounts for about 85% of all lung cancers and is itself divided into the main subgroups of squamous cell carcinoma (SCC), adenocarcinoma (ADC) and large cell carcinoma [1, 2]. The morphology of those subtypes is distinguished by immunohistological testing. The histology of patients whose measurements have been used in this study (SCC, ADC and adenosquamous carcinoma as combination of both) shall be discussed briefly in the following, as they show very different behavior in growth, metastatic seeding and response to treatment.

### Adenocarcinoma

ADC is the most frequent type of lung cancer located mainly in the lung periphery. Its histology is very heterogeneous but several markers have been established to identify this type of lung cancer. TTF1 has been shown to be expressed in about 80% of primary lung ADC [3]. In the complementary 20% it is useful to examine the expression of Napsin A that also proves the preparation to be part of the primary ADC of the lung [4]. It has been shown that mutations of KRAS correlate with worse prognosis and worse response to chemotherapy [5–7]. Targeted therapies of inhibitors of EGFR, EML4-ALK and ROS1 though show better efficiency for alterations in the respective genes, proteins and pathways [8–10]. The identification for markers of successful treatment is current scientific work [10].

### Squamous cell carcinoma

SCC is the second-most common type of lung cancer accounting for about 17% of lung cancer diagnoses. The incidence of SCC is strongly associated with cigarette smoking with a higher ratio for men than for women [11]. To distinguish SCC from SCLC immunohistochemical analysis of the markers Chromogranin A, Synaptophysin and CD56 is helpful. Molecular markers to identify differentiations within the group of SCC are cytokeratins such as CK5/6, CK14 and proteins such as p40 and p63. Morphologically SCC shows cornification and/or intracellular bridges and its proliferation rate is considered as high compared to healthy tissue [12]. Adenosquamous carcinoma (ASC) is considered a tumor that shows components of both SCC and ADC. The survival prognosis of patients with ASC is worse than those of SCC or ADC alone [13].

Lung cancer in general is very likely to metastasize and about 90% of cancer deaths do not occur due to the primary tumors but due to its metastases. Limited technical possibilities are the reason why most metastases are not identified at primary diagnosis [14–16]. Non-identified metastases might still influence therapeutic outcomes as decisions on treatment are also formed depending on the existence of metastases and the corresponding properties [14, 17–19]. The treatments of choice including metastases as therapeutic targets are systemic therapies, such as chemo- and immunotherapy. For non-altered genes, proteins and pathways as presented before, usually an immunotherapy based on targeting the PD-1 pathway is administered directly (so-called First-Line therapy) or subsequently to a prior platinum-based chemotherapy (so-called Second-Line therapy) [20]. The details for those mechanisms are discussed in the modeling section of this study.

## Mathematical model

### Primary tumor growth

Different ways to estimate tumor growth have widely been examined in various research works. Knowing the growth of a tumor over time not only gives predictive power and knowledge on the severeness of a cancerous disease but also provides possibilities of treatment optimization. It was found that for different tumors there is no ‘universal growth law’ to describe the tumor growth in a reliable way for clinical practice [21]. However, the Gompertz model, originally introduced in [22] and firstly applied to tumor measurements in [23], describes the tumor growth process in an easy but astonishingly efficient way for human tumors, in contrast to exponential growth describing experimental tumors [24]. The advantage of the Gompertz model is its initial exponential growth phase that slows down by exponential decay of its parameter to reach an asymptotical value for *t*→*∞*. Let this asymptotical value be denoted as *K*, interpreted as the tumor carrying capacity and let *x*(*t*) denote the tumor size at time *t*≥0. Then $x(t) \xrightarrow { t \to \infty } K$ by definition and by using *x*_0_>0 as the initial tumor size the Gompertz model reads $\frac {dx(t)}{dt} = r \exp (-at) x(t)$ with *x*(0)=*x*_0_. The parameters *r* and *a* can be interpreted as the initial exponential growth rate and its decay factor, respectively. Assuming the ‘one renegade cell’ theory, i.e. assuming the cancer’s origin to be one mutated cell, we have *x*_0_=1 [14, 25]. We can also directly formulate the Gompertz equation depending on the tumor carrying capacity *K* (computation can be found in the appendix): another possible formulation of the Gompertz growth law is to use *K*= exp(*r*/*a*) for *x*_0_=1, such that the system reads

1 $$ {\displaystyle \begin{array}{cc}\frac{dx(t)}{dt}& =a\ln \left(K/x(t)\right)x(t)\\ {}x(0)& =1\end{array}} $$

For further analysis, the value of *K* will be fixed to an amount of 10^12^ cells known from observations [26]. These ordinary differential equations (ODEs) for the Gompertz growth can be solved to derive analytical solutions for *x*_0_=1:

2 $$ x(t) = \exp \left(\frac{r}{a} \left(1-\exp (-at) \right) \right) = K^{1 - \exp (-at)}  $$

We can compute the age of a tumor *T*, which is the time a tumor of size *x*_0_=1 needs to grow to size *x*(*T*), by solving Eq. (2) for time as a function depending on size *x*. Let *T*(*x*) be this function, then

3 $$ T(x) = -\frac{1}{a} \ln \left(1 - \frac{a}{r} \ln \left(x \right) \right) = - \frac{1}{a}\ln \left(1 - \frac{\ln \left(x \right)}{\ln \left(K \right)} \right)  $$

The clinical characterization of the tumor volume doubling time (TVDT) originally introduced for exponential tumor growth [27] can also be applied to the Gompertz growth law. The TVDT is defined as the time a tumor of size *x*>1 takes to grow to size 2*x* and can be estimated clinically [28]. Using Eq. (3) one receives

4 $$ {}\begin{aligned} TVDT(x) &= T(2x) - T(x) \\& = -\frac{1}{a} \ln \left(\frac{a \ln \left(2x \right)}{a \ln \left(x \right) - r} - \frac{r}{a \ln \left(x \right) - r} \right)\\ & = - \frac{1}{a} \left(\ln \left(1 - \frac{\ln \left(2x \right)}{\ln \left(K \right)} \right) - \ln \left(1 - \frac{\ln \left(x \right)}{\ln \left(K \right)} \right) \right). \end{aligned}  $$

### Therapy dynamics: chemotherapy

As there are close to no measurements of untreated tumors in humans, a comparison of model formulations to clinical measurements is only possible when including the corresponding therapy dynamics into the model. The general principle of chemotherapeutic agents is formed of cytostatic and/or cytotoxic effects on different pathways of cells, in the ideal case solely on cancerous ones or with minimal side effects on healthy cells [29, 30]. As adverse effects in chemotherapy application are usually very common, many different combination therapies of classical drugs and newer targeted therapy methods have been used as a standard of care for different cancerous diseases [30]. Still, the common theory behind the quantitative effects of chemotherapy is originally formed by the so-called ‘log cell kill’ theory [31, 32]. It states that an applied chemotherapy dose kills a constant fraction of tumor cells independent of the actual tumor size during a certain fixed amount of time. Experimental regimes have shown this behavior in mice as long as the mentioned exponential growth has been identified, in stark contrast to human cancer settings [33]. For human tumors, it was suggested that the chemotherapy exhibits effects in tumor regression proportional to the growth rate of an untreated tumor of this size and that the tumor size also depends on the integrated drug effect during the course of actual treatment [34, 35]. This allows for an explanation of refractory effects termed as ‘kinetic resistance’ that are widely observed in clinical chemotherapy applications on tumors reaching a small size [24]. This kinetic resistance is an important driver of therapy outcome.

To account for these refractory effects in a simpler way we introduce the dynamics of chemotherapy as follows. Let $\mathbbm {1}_{C_{i}}(t)$ denote the characteristic function returning a logical value that indicates the application of a chemotherapeutic drug *C*_*i*_ at time *t*≥0. This idea put into a mathematical formulation yields

5 $$ \begin{aligned} \mathbbm{1}_{C_{i}}(t) = \left\{\begin{array}{ll} 0 \text{, if chemotherapy is not applied}\\ 1 \text{, if chemotherapy is applied} \end{array}\right. \text{ at } t \geq 0 \text{ using pharmaceutical}\ i. \end{aligned}  $$

Further assume that on days without chemotherapy, the concentration of chemo-therapeutic drugs within the body and thus their effect is negligible. This is realistic for chemotherapeutic drugs, as the half-life of these pharmaceuticals is relatively small and lies in ranges of several hours. By this definition $\mathbbm {1}_{C_{i}}(t)$ has a finite amount of jump discontinuities if both the application time of chemotherapeutic drugs and the amount of applications of the respective drug is measurable but finite. Let *μ*_*i*_(*t*) denote the fractional effect of chemotherapy administration of drug *i* on the tumor size. The refractory effect of drug administration can be expressed based on an idea of [36] as a change $\frac {d \mu _{i} (t)}{d t}$ reducing the effects in a constant fraction $\mu _{i}^{*} \geq 0$ when the drug is applied, accounting for a fraction of the tumor to show resistance towards the drug, using *μ*_*i*_(0)=*μ*_*i*,0_≥0.

6 $$ \frac{d \mu_{i} (t)}{dt} = \left\{\begin{array}{ll} 0 & \text{, if } \mathbbm{1}_{C_{i}} (t) = 0\\ -\mu_{i}^{*} \mu_{i} (t) & \text{, if } \mathbbm{1}_{C_{i}} (t) = 1 \end{array}\right.  $$

Taking these effects for drug application into account for the growth dynamics of a tumor one has^1^(We neglect the tumor’s growth rate in the case of active treatment as the application time of a chemotherapeutic drug is rather short and they feature half-lives of just a few hours):

7 $$ \frac{dx(t)}{dt} = \left\{\begin{array}{ll} a \ln (K/x(t)) x(t) & \text{, if } \mathbbm{1}_{C_{i}}(t) = 0\\ -\mu_{i} (t) x(t) & \text{, if } \mathbbm{1}_{C_{i}} (t) = 1. \end{array}\right.  $$

This approach considers the emergence of resistance towards treatment with respect to the already approved therapeutic windows of the applied drugs. The choice of $\mu _{i}^{*}$ therefore suggests the emergence to only be proportional to the application times of the chemotherapeutic drug. In theory, the approach could be extended to have the emergence to depend also on the administered dose, i.e. the fraction $\mu _{i}^{*}$ is a function of the given dose per time. Deviations from the approved dosages are not in this work’s focus.

### Therapy dynamics: immunotherapy

The growth of tumors and their metastatic spread is not only determined by cell characteristics but also influenced by the immune system [37]. Immunologic approaches have become an inherent part in tumor therapy within the past decades and contribute to the antitumoral activity of the immune system [38, 39]. In general, an immunotherapeutic treatment can be divided into four major subgroups: the active non-specific immunotherapy (treatment e.g. via cytokines), the active specific immunotherapy (vaccines), the passive immunotherapy (monoclonal antibodies) and approaches to block immune escape mechanisms (e.g. CTLA-4) [40]. In the following, we will focus on the application of certain immune checkpoint inhibitors, that usually limit the activation of the immune system. Tumor cells can manipulate and dysregulate immune checkpoints to modify the T-cell activity and suppress an immune response [41, 42].

The PD-1-pathway as a specific immune checkpoint can regulate the T-cell activity in the effector phase of the immune response. Usually, the activation of PD-1 serves as a downregulation of T-cell activity in the peripheral tissue to prevent collateral damage during an immune response [42, 43]. However, cancer cells can manipulate the PD-1-pathway by expressing the PD-1-Ligands PD-L1 and PD-L2. Those ligands bind on the T-cell’s PD-1 receptor and inactivate the T-cell to block the immune response towards the tumor cells [41, 42, 44, 45].

PD-L1 is widely expressed in many different cancer types in varying amounts which has made it an attractive target for novel treatment approaches [46]. Anti-PD-1 and Anti-PD-L1 antibodies have become standard treatments for different cancer types in the last few years showing significant clinical benefits in patients with advanced stages of cancer [47]. An overview over the currently approved therapeutics is given in Table 1.

**Table 1:** Monoclonal antibodies

Antibody	Type	Year of approval (FDA)	Molar Mass *M*_*i*_ [kDa]	Approved Dosages	Half-life $t^{1/2}_{i}$ [d]	Sources
Atezolizumab	humanized	2016	145	1200mg q3w	27	[70, 71]
Avelumab	human	2017	143	800mg q2w	6.1	[72, 73]
Durvalumab	human	2017	146	10mg/kg q2w	18	[74, 75]
Nivolumab	human	2014	146	240mg q2w or 480mg q4w	26.7	[76, 77]
Pembrolizumab	humanized	2014	146	200mg q3w or 400mg q6w	22	[78, 79]
Cemiplimab	human	2018	144	350mg q3w	19.4	[80, 81]

Currently approved monoclonal antibodies used as immunotherapeutics targeting the PD-1 pathway. The application cycle length *l* used in the model is determined from the dosage, e.g. the expression “q3w” reads “every three weeks”, thus *l*=3

As those antibodies show half-lives much higher than those of the chemotherapeutic drugs modeled before, we have taken into account pharmacokinetic effects. Let *c*_*i*_(*t*) denote the concentration of drug *i* at time *t*≥0 measured in [amount of molecules per body volume]. Assume this volume to be constant for any individual patient due to missing data. The concentration’s dynamic at a given time point *t* of an immunotherapeutic pharmaceutical *i* is assumed to depend on the state of therapy. While the drug is applied, administration of a dose *d*_*i*_ measured in [mg per body volume per time] increases the concentration depending on the molar mass *M*_*i*_ of the drug and the Avogadro constant *N*_*A*_. The clearance depends on the drug’s half-life $t^{1/2}_{i}$ and takes place throughout the whole time course. The pharmacokinetic equation therefore reads

8 $$ \dot c_{i}(t) = \left\{\begin{array}{ll} -\left(\frac{\ln (2)}{t^{1/2}_{i}}\right) c_{i} (t) & \text{, if } \mathbbm{1}_{I_{i}}(t) = 0\\ -\left(\frac{\ln (2)}{t^{1/2}_{i}}\right) c_{i} (t) + \frac{N_{A}}{M_{i}} d_{i} & \text{, if } \mathbbm{1}_{I_{i}}(t) = 1 \end{array}\right.  $$

The characteristic function of immunotherapy is analogous to Eq. (5) used for chemotherapy and reads

9 $$ \begin{aligned} \mathbbm{1}_{I_{i}}(t) = \left\{\begin{array}{ll} 0 \text{, if immunotherapy is not applied}\\ 1 \text{, if immunotherapy is applied} \end{array}\right. \text{ at } t \geq 0 \text{ using pharmaceutical} i. \end{aligned}  $$

The immunotherapeutic drug is applied in cycles of a certain length *l* (cf. Table 1). This means that after one application of a daily dose, the time to the next application will be *l* time units, e.g. measured in days. To simplify model analysis the quasi steady state assumption may be applied to determine the steady state drug concentration that the simulation will periodically meet. Let this value be denoted as $c_{i}^{st}$. The applied mean drug dose over the whole application time $\tilde d_{i}$ can be computed as the application time related mean value $\frac {d_{i}}{l}$. Then the steady state value $c_{i}^{st}$ can be computed as

10 $$ c_{i}^{st} = \frac{N_{A}}{M_{i}} \tilde d_{i} \frac{t^{1/2}_{i}}{\ln (2)}.  $$

The quantitative effects of immunotherapy are not yet clearly resolved [48–50]. We therefore assume the immunotherapeutic drugs to show a behavior of a Hill-Langmuir equation with first order Hill coefficient in its efficiency towards cancer cells, using $c^{50}_{i}$ as the drug concentration necessary to show half of the efficiency. The parameter *χ* describes the per time amount of cancer cells destroyed by the direct application of one single molecule. The amount of cancer cells is still denoted by *x*(*t*), the pure growth dynamic stays as a Gompertz equation in the type of Eq. (1). The overall growth dynamic of cancer cells under immunotherapy therefore can be formulated as

11 $$ \frac{dx(t)}{dt} = a x(t) \ln \left(\frac{K}{x(t)} \right) -\chi \frac{c_{i}(t) x(t)}{c^{50}_{i} + c_{i}(t)}.  $$

### Metastatis growth and spread

Metastases and their treatment’s consequences are the strongest contributor to cancer-related deaths [51]. They form by cancer cells leaving the primary tumor mass and moving to distant sites within the human body via the blood and lymphatic vessels [14]. After extravasation out of the vessels into surrounding tissues those metastatic cells construct tumoral microenvironments and *micrometastases* to perform angiogenesis and proliferation [52]. The daughter tumors resulting from this so-called *colonization* are referred to as metastases [14]. Even though only a minority of such metastatic cells survives the travel through the body as of immune system reactions and other defense mechanisms, a diagnosis of metastases is directly linked to significantly lowered survival times [51, 53]. The crucial point is that, even when primary diagnosis has found only a few metastases in a patient’s body, there are usually hundreds or thousands of micrometastases widely spread over numerous tissues in the body [15, 16]. Their detection is very challenging if not even impossible with current techniques [14]. The total metastatic burden in a patient’s body does not only influence treatment options, its appraisal is of high importance for the outcome as well [17, 54].

Mathematically, the approaches on improving the understanding of cancer metastasis and estimating their development have been numerous, while population formulations have been a minority amongst them. Originally introduced by [55], a function *ρ*(*x*,*t*) formulated in a transport equation interprets as size-structured density of metastases. The function value *ρ*(*x*,*t*) gives the density of tumors of size *x*≥1 at time *t*≥0 measured in number of cells. The primary tumor dynamics can be introduced in this population approach with an initial condition *ρ*(1,0)=1 and *ρ*(*x*,0)=0∀*x*>1 corresponding to the cancer’s origin to be one single mutated cell as introduced earlier. Using the individual tumor’s growth rate *g*(*x*,*t*) as stated above for either treatment (a joint treatment is clinically not applicable) and the natural growth *g*(*x*,*t*)=*a* ln(*K*/*x*(*t*))*x*(*t*) in absence of any treatment, the following dynamics are further assumed:

12 $$ \frac{\partial \rho(x,t)}{\partial t} + \frac{\partial g(x,t) \rho(x,t)}{\partial x} = 0  $$

and a boundary condition to introduce newly formed metastases of cell size one

13 $$ g(1,t) \rho(1,t) = \int_{1}^{\infty} \beta(\tilde x,t) \rho (\tilde x,t) d\tilde x  $$

Here we integrate over $\tilde x$ to clarify that it is all sizes within the distribution that we consider in the boundary condition, not only the tumor size *x* that is denoted in the transport equation. The dissemination rate $\beta (\tilde x,t) := m \tilde x^{\alpha }$ describes the steps of the metastatic cascade necessary to take place for successful metastasis formation [15]. It is measured in the amount of formed metastases per unit time, as *m* denotes the colonization coefficient and *α* describes the fractional dimension of the tumor. For a tumor of spheroid shape, this parameter equals 2/3 as fraction of surface by volume. Another possible interpretation focuses on the probability of tumor cells to metastasize: for *α*=0 the dissemination rate turns out to be a constant cell pool (e.g. stem cells) whereas *α*=1 indicates equal probability for all tumor cells to metastasize. Values 0<*α*<1 are seen as the geometric disposition of cells potentially metastasizing, while the parameter *m* can be interpreted as the per day per cell probability for tumor cells to overcome every single step of the metastatic cascade [56]. By construction, it is assumed that all metastases show the same growth and seeding behavior as the primary tumor [57]. This includes the equal carrying capacity *K* for each individual metastasis.

To include the previously formulated tumor growth and therapy methods, one introduces formulations on the stepwise time course depending on therapy-free growth or therapy effects on the respective tumor with help of the Eqs. (1), (7) and (8). This now can be used for the rate *g*(*x*,*t*) in Eq. (12) depending on the given desired therapy schedule. As those need to be defined previously and patient-individually, it is pure simulation to compare therapy outcomes of different schedules. Comparisons with clinical data can be found in the following section.

Of further interest are the number of metastases, be them visible or not yet detectable with imaging techniques. The cumulative size distribution of visible tumors is the number of tumors larger than a certain size threshold *s*_*vis*_ that depends on the resolution of the clinically used techniques. The quantity reads

14 $$ N_{vis} (t) = \int_{s_{vis}}^{\infty} \rho (\tilde x,t) d\tilde x  $$

whereas the total tumor burden (the sum of metastases and the primary tumor) can be defined as

15 $$ N_{0} (t) = \int_{0}^{\infty} \rho (\tilde x,t) d\tilde x.  $$

## Materials & methods

### Clinical data

The patient data examined in the following contain patients with non-small cell lung cancer (NSCLC) and were collected from volumetric measurements of primary tumors and metastases of patients with adenocarcinoma and adenosquamous carcinoma with different histologies (see Table 2 for details). All patients were routinely treated in the Clinic of Pneumology, Thoracic Oncology, Sleep and Respiratory Critical Care of the Klinikverbund Allgäu in Germany. Data use was approved by the ethic commission of BLAEK (Ethik-Kommission der Bayerischen Landesärztekammer), reference number 19021. The volumetric measurements originate from routinely acquired CT slices and computations of the secondary appraisal environment *syngo.CT* Lung Computer Aided Detection (CAD) workflow of Siemens Healthineers, provided in the *syngo.Via* VB40 framework. Prototype versions of this Lung CAD system provided a sensitivity of about 87% for detecting lung nodules also identified by three out of four radiologists as well as a sensitivity of about 89% for detecting lung nodules identified in radiologist’s consensus [58]. More modern versions trained on higher amounts of data and improved underlying detection algorithms have been estimated to show a sensitivity of correctly identifying lung nodules at a rate of about 97% [59]. The false positive-rate in these examinations has shown to range between 3.0 and 2.8 per examination on average for 75% and 100% consensus of radiologists respectively [58] as well as 3.7 and 1.9 per examination on average when searching for all nodules or nodules of sizes larger than 50 mm^3^ respectively [59]. Those volumetric data were rescaled into measurements [amount of cells] by using the conversion rule 10^−3^ml =1*mm*^3^=10^6^ cells [60].

**Table 2:** Patient-specific clinical parameters

Patient	KE-01	KE-02	KE-03
Sex	F	M	M
Histology	Adenosquamous Carcinoma	Adenocarcinoma	Adenocarcinoma
Molecular Pathology	EGFR-, PD-L1 5, CK7+, TTF1+,	EGFR-, ALK-, KRAS-, BRAF-,	EGFR-, ALK-, KRAS-,
	p63+, Chromogr-, Syn-	PD-L1 50, Ros1-	BRAF- PD-L1 5
TNM Classification	cT4 cN2 cM1b	cT1b pN3 M1a	cT4 cN3 cM0
ECOG-PS	1	0	0
Size of PT at primary diagnosis [ml]	240.74	14.27	71.75

The three patients with NSCLC examined in this study were routinely treated with a prior Cisplatin/Pemetrexed Chemotherapy and/or 1L/2L immunotherapy (Pembrolizumab or Nivolumab), see “Results” section 4 for further details. The molecular pathology features diverse histological testing of tumor markers as follows: *EGFR*: Epidermal Growth Factor Receptor, *PD-L1*: Programmed Death Ligand 1, *CK7*: Cytokeratin 7, *TTF1*: Thyroid Transcription Factor 1, *p63*: Tumor Protein 63, *Chromogr*: Chromogranin A, *Syn*: Synaptophysin, *ALK*: EML4-ALK fusion protein, *KRAS*: Kirsten Rat Sarcoma, *BRAF*: rapid accelerated fibrosarcoma (B-Type), *Ros1*: rather often translocated in sarcoma. The sign “+” or “-” indicates a positive respective negative test result for those gene translocations and upregulations. The connection of mentioned markers to the diagnosis of tumor histology is explained in the first section

## Results

### Model implementation and fitting

The volumetric measurements contain time course dynamics of the tumors identified by *syngo.CT*. As it is in general not distinguishable purely from clinical observations whether re-seeding occurs between the primary tumor and its metastases, we employed Iwata’s modeling approach [55] introduced in Eqs. (12) and (13) and calculated the total tumor burden at the respective CT examination date. In this way, the possibility of re-seeding is accounted for to combine the modeling framework results to clinical data. Also, it is not only single cells that may disseminate in the metastatic process but also cell packages that might form new metastases. In the modeling framework, these cell packages are distinguished into their individual cells. To meet these calculations, the implementation of the model was performed with a discretization using a method of solving the PDE on its characteristics in MATLAB^*Ⓡ*^ including the primary tumor dynamics into the density via the initial condition *ρ*(1,0)=1 and *ρ*(*x*,0)=0∀*x*>1. In the discretized form, this condition satisfies the assumption of one single existing primary tumor of size one cell at reference time *t*=0. Depending on the therapy schedule, the growth function *g*(*x*,*t*) had the form of Eq. (1) for no treatment, the form of Eq. (7) for the time interval of chemotherapeutic treatment and the form of Eq. (11) for the time interval of immunotherapeutic treatment. The immunotherapeutic treatment’s drug concentrations were solved step-wise using the pre-defined ode45-framework of MATLAB^*Ⓡ*^ on a daily concentration amount. The ode45 framework is based on an explicit Runge-Kutta (4,5) formula [61–63]. The parameters necessary to describe the metastases’ time course were fitted from the calculated total tumor burden model output of Eq. (14) using the pre-defined MATLAB^*Ⓡ*^-framework fmincon [64] using carefully chosen applications of switches between the sqp and interior-point algorithms on different initial parameter space values minimizing least squares towards the total tumor burden data points. Diverse starting points in reasonable parameter intervals were chosen and biologically relevant fitting results were identified. The standard errors have been computed as the square root of the diagonal entries of the inverse of the resulting Hessian matrices. As clinical history was different for every single patient, this had to be established individually for any patient.

### Individual parameters

**Fig. 1:**
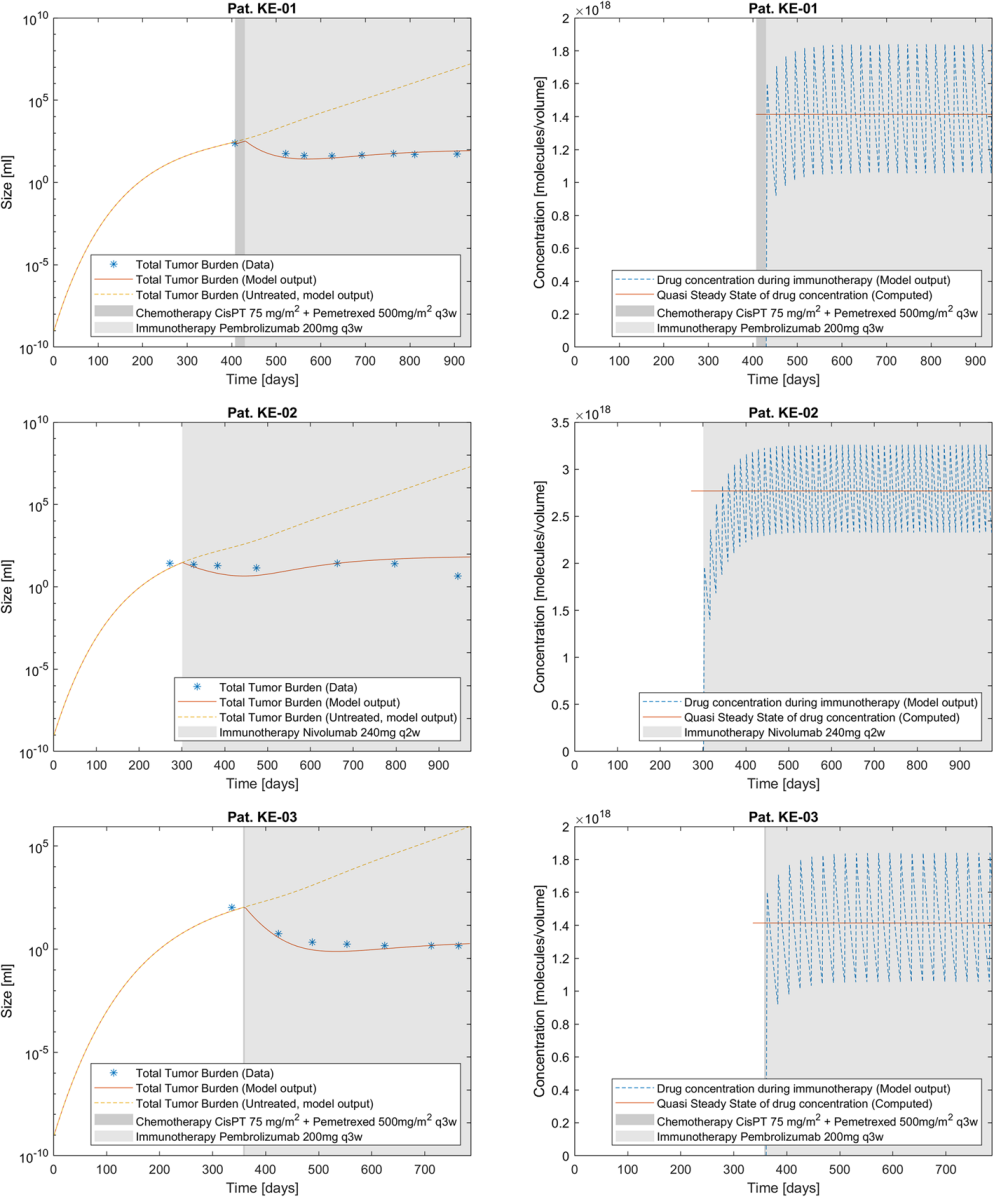
Patient dynamics: total tumor burden and immunotherapeutic drug concentration. The model outputs for tumor burden under therapy compared with therapy-free simulations and data (left column) as well as model output for immunotherapeutic drug concentration (right column) for the three patients (with respective rows) evaluated in this study. The respective clinical detection limit *s*_*vis*_ was determined as the minimum volume data point of this patient’s total treatment

The fit calculations have led to parameter values presented in Table 3. The therapeutic time course, the treatment schedules of the patients and the model predictions on the total tumor burden resulting from the model implementation can be found in Fig. 1 with the corresponding computed immunotherapeutic drug concentrations under application (cf. Eq. (8)).

**Table 3:** Patient-specific model parameters

	Patient	KE-01	KE-02	KE-03	
Parameter	Explanation				Origin
*K*	Environmental carrying capacity [cells]	10^12^	10^12^	10^12^	fixed, [26]
*a*	Growth rate [1/day]	7.284·10^−3^	6.877·10^−3^	6.984·10^−3^	fitted
		(1.041·10^−3^)	(3.734·10^−3^)	(0.611·10^−3^)	
*m*	Colonization coefficient [1/cell 1/day]	1.635·10^−7^	1.984·10^−7^	2.738·10^−7^	fitted
		(0.157·10^−7^)	(3.816·10^−7^)	(0.490·10^−7^)	
*α*	Fractal dimension of tumor cells able to metastasize [-]	2/3	2/3	2/3	fixed, [55]
	Chemotherapeutic drug in use	*CisPT/Pemet. (1L)*	-	*CisPT/Pemet. (1L)*	Data
*μ*_0_	Initial chemotherapy efficiency [-]	0.237 (1.000)	-	0.081 (0.917)	fitted
*μ*^∗^	Refractory effect under chemotherapy application [-]	0.132 (1.000)	-	0.132 (1.321)	fitted
	Immunotherapeutic drug in use	*Pembr. (2L)*	*Nivol. (1L)*	*Pembr. (2L)*	Data
*χ*	Immunotherapeutic effect under application [1/day]	0.067 (0.096)	0.499 (0.078)	0.088 (0.011)	fitted
$c_{i}^{50}$	Drug concentration of immunotherapeutic drug	1.012·10^16^	1.010·10^16^	1.009·10^16^	fitted
	for half-maximal response [molecules per volume]	(0.142·10^16^)	(0.089·10^16^)	(0.020·10^16^)	
$c_{i}^{st}$	Drug concentration of immunotherapeutic drug	1.41·10^18^	2.77·10^18^	1.41·10^18^	estimated,
	in serum steady state [molecules per volume]				cf. Eq. (10)
*T*	Age of primary tumor at diagnosis [d]	407.1	272.2	336.5	estimated,
					cf. Eq. (3)
*TVDT*	Tumor volume doubling time at diagnosis [d]	91.6	25.9	43.7	estimated,
					cf. Eq. (4)

*CisPT/Pemet.* = Combination therapy of Cisplatinum and Pemetrexed. *Pembr.* = Pembrolizumab. *Nivol.* = Nivolumab. *(1L)* = First-line therapy. *(2L)* = Second-line therapy. The values in brackets indicate the corresponding standard errors

### Findings

Similarly as argued by [56] our estimated proliferation rates *a* yield corresponding tumor cell cycle lengths of $\frac {ln(2)}{a}$ hours, i.e. those lengths are indirectly estimated to lie between 93.1 and 100.8 hours. These values are realistically close to cycle lengths of observed values between two to four days [65]. Also, tumor volume doubling times in the range of the calculated values (cf. Table 3) have been reported clinically [28]. The chosen Gompertz equation is therefore considered appropriate for the patient’s growth dynamics. After successful estimation of the underlying parameters from the available data, the model is able to reconstruct the time course of clinical history of the patients, that is, the underlying seeding and growth dynamics of the primary tumors and corresponding metastases that have been diagnosed throughout the treatment of the patients.

**Fig. 2:**
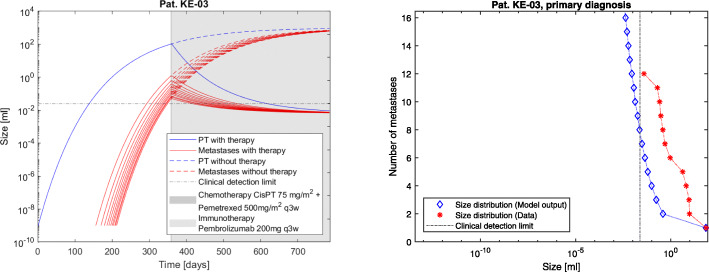
Patient KE-03. The model output for primary tumor and metastasis dynamics on an individual level under therapy compared with therapy-free simulations and data (left) as well as model output for the tumor size distribution at day 315 after primary tumor initiation, which is the patients primary diagnosis (right). Cf. Table 1 for the patients clinical parameters. For better readability, we have plotted the dynamics for the largest metastases only. The corresponding total mass depending on time including the non-plotted metastases is shown in Fig. 1

**Fig. 3:**
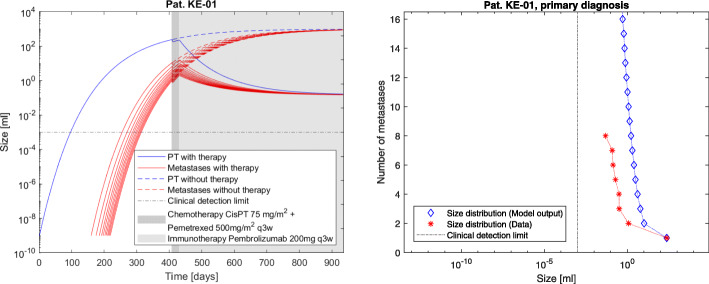
Patient KE-01. The model output for tumor dynamics on an individual level under therapy compared with therapy-free simulations and data (left) as well as model output for the tumor size distribution at day 407 after primary tumor initiation, which is the patients primary diagnosis (right). Cf. Table 1 for the patients clinical parameters. For better readability, we have plotted the dynamics for the largest metastases only. The corresponding total mass depending on time including the non-plotted metastases is shown in Fig. 1

**Fig. 4:**
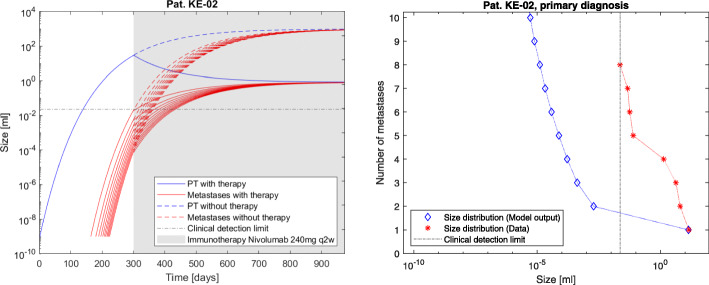
Patient KE-02. The model output for tumor dynamics on an individual level under therapy compared with therapy-free simulations and data (left) as well as model output for the tumor size distribution at day 272 after primary tumor initiation, which is the patients primary diagnosis (right). Cf. Table 1 for the patients clinical parameters. For better readability, we have plotted the dynamics for the largest metastases only. The corresponding total mass depending on time including the non-plotted metastases is shown in Fig. 1

The model advantage is to determine the individual tumor dynamics under time course and therapy application by examining the metastatic density at fixed time points. The resulting simulations are shown in Figs. 2, 3 and 4. The computations conclude that at the time of primary diagnosis the patients theoretically had 185, 45 and 119 tumors including micrometastases while the measurement data acquired in routine treatment showed 8, 8 and 12 (visible) metastases respectively. The total tumor burden volumes at primary diagnosis could be calculated correctly, as shown in Fig. 1. Clinical distinction of single metastases is not entirely possible, as different seeded metastases could have grown together to form one single clinically traceable metastasis. Therefore the focus on the total metastatic mass as clinical measurement was chosen for this approach and explains deviations of the measurements from the densities of the simulation.

**Fig. 5:**
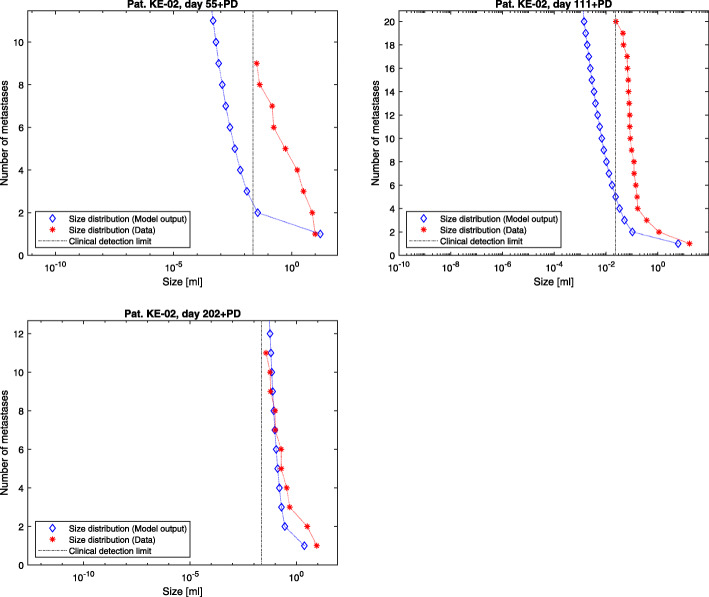
Patient KE-02. Tumor size distributions for the examinations with CT material following primary diagnosis after 55 (upper left), 111 (upper right) and 202 (lower) days. The model output distributions are compared to the data points of the corresponding days

**Fig. 6:**
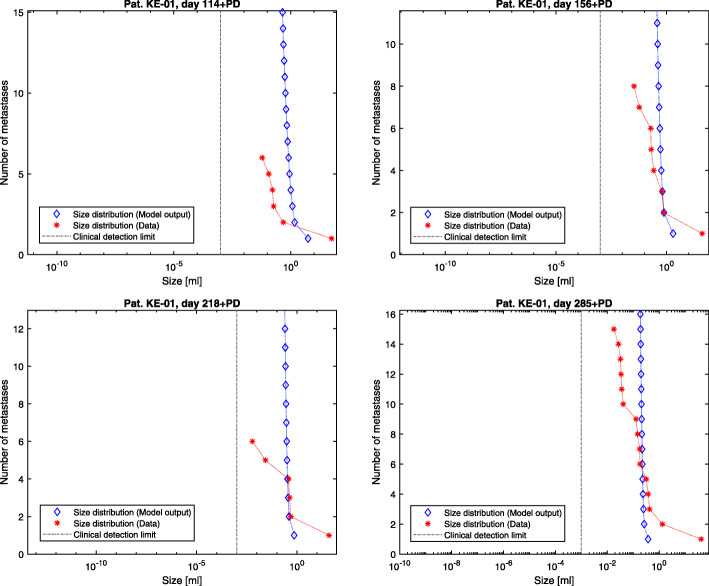
Patient KE-01. Tumor size distributions for the examinations with CT material following primary diagnosis after 114 (upper left), 156 (upper right), 218 (lower left) and 285 (lower right) days. The model output distributions are compared to the data points of the corresponding days

**Fig. 7:**
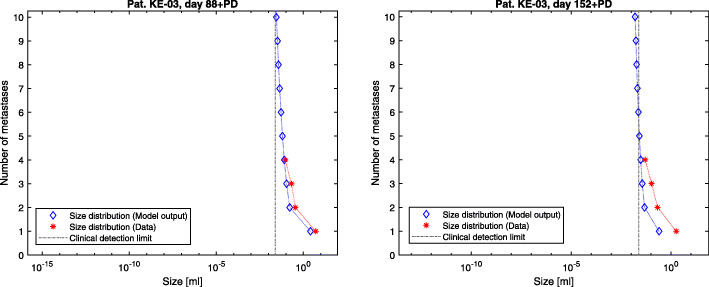
Patient KE-03. Tumor size distributions for the examinations with CT material following primary diagnosis after 88 (left) and 152 (right) days. The model output distributions are compared to the data points of the corresponding days

The presented model framework does not only show the possibility to retrace the clinical history of patients but also to predict the outcome of a vast variety of different hypothetic treatment regimes on primary tumor and metastases on an individual patient level given the above stated assumptions. The model output for the metastases’ dynamics are referred to in Figs. 2, 3 and 4 compared to the what-if scenario of untreated tumors showing the same dynamics. Figures 5, 6 and 7 show the time course of metastatic densities compared to acquired data points from all three patients.

**Fig. 8:**
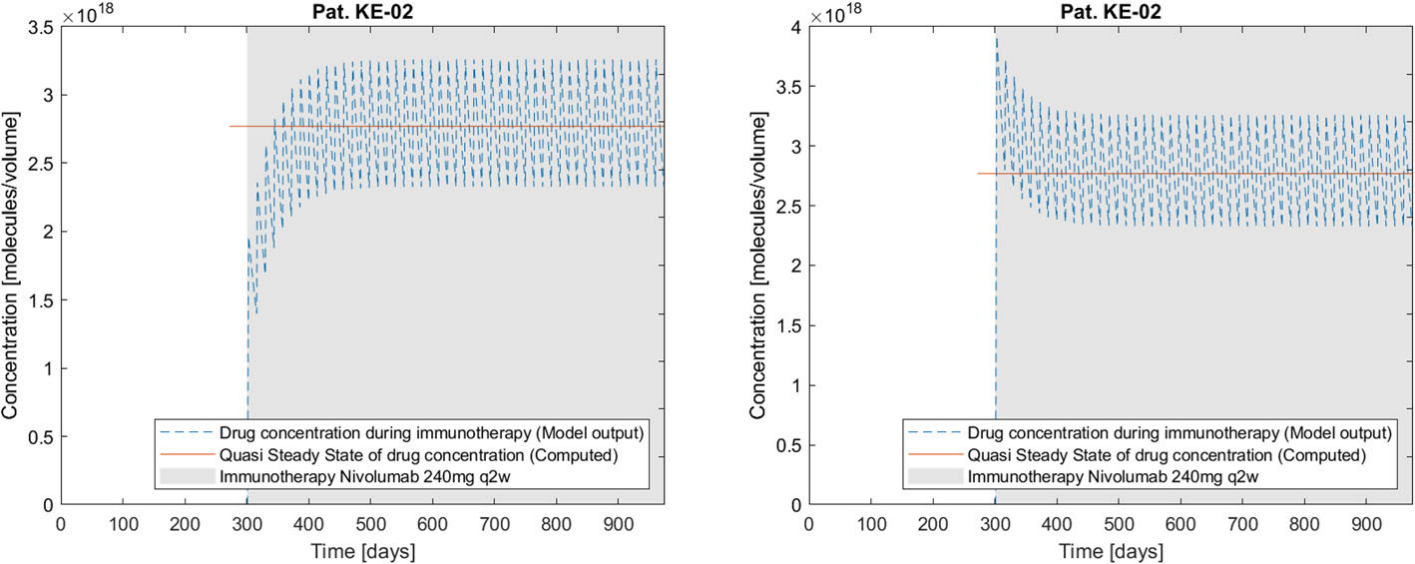
Patient KE-02. The model output for immunotherapeutic drug concentration under therapy for the approved 240mg q2w regimen (upper left) compared with immunotherapeutic drug concentration in a deviating regimen starting with the doubled dose (480mg) for the initial therapy cycle and subsequent 240mg q2w treatment (upper right). The doubled-dose-regimen shows better response than the standard schedule. The data points are the same as shown in Fig. 4, while the doubled-dose-regimen shows an improvement in reducing the metastatic mass by about 0.4%

Interestingly the model was able to reconstruct parameter values that are reasonable when taking into account the underlying biological factors. It was shown that for tumors expressing higher amounts of PD-L1, their proliferation and malignancy is also increased [66]. This is supported by a lower estimated tumor volume doubling time of patient KE-02 compared to the two other patients, as the patient also shows a higher PD-L1 score. A fourth patient KE-04 (data not shown) treated with tyrosine kinase inhibitors has been fitted to the same model, to check the model’s predictive power for differing drug action mechanisms. The extracted growth rate *a* for this patient was larger than of those patients presented in the study at hand. This is easily explained by patient KE-04’s *EGFR*-del9 mutation, as this features drastically larger tumor proliferation. The three patients presented before do not show this mutation. The model was therefore successful to quantify effects of certain clinical parameters on tumor growth and metastatic density as well as treatment outcomes for different immunotherapeutic agents. Assuming the parameters to behave equally for differing treatment regimens, the model framework can be used to determine outcomes of adjusted dosages (cf. Fig. 8 and the “Discussion” section).

## Discussion

The study presents a novel modeling framework describing individual patient-based densities of tumors in metastatic cancerous diseases. The framework is based on systems of ordinary and partial differential equations including the dynamics of therapeutic schedules of chemo- and immunotherapy. The model was tested on measurement series of three different patients with NSCLC to acquire model parameters via fitting. These parameters and their relations might contribute to a better understanding of the disease outcome for different diagnostics and their behavior under therapy. The study at hand is of course not explaining these relations solely relying on three patient measurement series but uses them to prove functioning of the concept.

The model was fitted for patients with metastases in the lung assuming the growth kinetics of metastases to be identical to the one of their primary tumor. It is observed that the growth parameters of primary NSCLC stay identical even for its brain metastases [56], but no general rule has been established yet to confirm this hypothesis. If this hypothesis was falsified the model could also be used for metastases that show different growth kinetics than their primary tumor. This could be done by introducing different metastatic densities *ρ*_*i*_ for *i* different groups of metastases sharing the same growth kinetics and formulating the presented approach for each *ρ*_*i*_ individually.

The model accuracy was chosen appropriately to the available and acquired data, the framework returned parameter relations of the different patients that were also observed in clinical settings. The differing drug dosage assumed by us is solely focusing on antitumoral effects, not on adverse or toxic effects on the patient. By relying on drug administrations in permitted regimens it is secured that the given dosages lie within approved therapeutic windows. Further modeling needs to take into account the patients’ performance to draw solid clinical conclusions. This could potentially allow to examine deviations from pregiven standardized therapy plans.

Widely performed treatments base on the goal of increasing the patients’ overall survival. For this, it might be helpful to consider the concept of early tumor shrinkage [48]. There are discussions whether higher doses of Nivolumab in shorter application intervals could be applied in the initial therapy stage to achieve this early tumor shrinkage by faster reaching the drug serum steady state and thus providing better responses [67, 68]. The tumor size distributions shown in Fig. 8 for patient KE-02 confirm this approach quantitatively. Another aspect is to optimize the dosage of Nivolumab, as it has been shown that low-dose Nivolumab could have comparable efficacy on cancer patients due to its flat dose-response relationship [50]. The flat relationship is mapped by the values of $c_{i}^{50}$, the drug concentrations yielding half-maximal response, that are by far lower than the body concentration steady states of the drugs when applied (cf. Table 3). Computations for patient KE-02 have shown that a half dosage yields half the steady state concentration value $c_{i}^{st}$ but reaching it after the same amount of administration cycles. A doubled dose in the very initial administration cycle would reach this steady state already from the onset of the treatment (cf. Fig. 8). The lowest dose showing sufficient antitumoral effects is yet to be determined and could be optimized using this framework.

Further, it has been shown that immunotherapeutic drugs provide survival benefits to patients irrespective to their PD-L1 score with reasons for these effects unknown [69]. Data analysis in terms of the presented model framework might give insights into clinical factors and patient characteristics that are responsible for these effects.

The fitted model parameters lie within reasonable intervals and are in ranges of the same order of magnitude for the presented measurements. Their accuracy could be improved with more data points within a longitudinal data set. Simulations based on these parameters show that the amount of the total tumor burden is mainly depending on the primary tumor and early formed metastases. The late disseminated metastases are of less importance in the therapy setting and might not contribute to the treatment outcome or the patient’s death [57]. This is also supported by clinical observations [26].

Certain important datapoints such as precise amount, position and size of identified tumors necessary for the calibration of the predictive framework have been generated by CAD algorithms commercially available using routinely acquired images. The preprocessing for the presented modeling approach included running these algorithms on the raw data sets, extracting resulting datapoints and transferring them for model calibration. In the clinics, the preprocessing is not done routinely. In the future, the preprocessing could be automated, allowing the model to be used in a more comprehensive way. The possibility to generate volumetric data is solely an issue of processing power, as existing clinically manufactured images are vastly available from routine diagnostics.

## Conclusions

This is, to authors’ knowledge, the first model to distinguish different therapy schedules in a mathematical model taking the dynamics of metastases into account. The model could give a foundation to establish mathematical models and predictive power into personalized medicine. To this day clinicians form therapy decisions based on the clinical history of the individual patient, his current status and a prediction based on impersonal/general study populations applying commonly used statistical methods. This framework allows to model and quantify customized and personalized estimates for future tumor development and long-term behavior under different treatment regimens even before making therapeutic decisions. In the future this could assist clinicians to generate and directly compare quantifiable therapy impacts and their outcomes allowing a qualified and quantified decision meeting personalized needs for future individual treatments.

The framework is very practical as it is based on deterministic equations and a minimum of parameters allowing it to be computable by any personal computer. Its practicability could be extended and implementation into daily clinical use could be simplified by automating preprocessing as mentioned earlier. The future significance of this model is attributed to its practical implementation and relevance in the field of personalized medicine promising comprehensive use.

Future work will focus on the evaluation of a larger patient database, the extension of the framework at hand to different medication regimens as well as the follow-up fitting of measurement series to improve the knowledge about modality and behavior of the model parameters and how to determine their ranges by histologies of primary diagnosis. Further, we expect future work to reduce the standard errors of the model parameters and to increase reliability of the model outputs by fitting on a density level instead of total tumor burden.

## Data Availability

The datasets used and analyzed during this study are available from the corresponding author on reasonable request. Declarations

## Footnotes

1 The log cell kill hypothesis could be modeled by having *μ*_*i*_≥0 constant and independent of time, i.e. $\frac {d \mu _{i}}{dt} = 0$.
